# The crystal structure and Hirshfeld surface analysis of 1-(2,5-di­meth­oxy­phen­yl)-2,2,6,6-tetra­methyl­piperidine

**DOI:** 10.1107/S2056989020005952

**Published:** 2020-05-05

**Authors:** Mustapha Tiouabi, Raphaël Tabacchi, Helen Stoeckli-Evans

**Affiliations:** a Institute of Chemistry, University of Neuchâtel, Av. de Bellevax 51, CH-2000 Neuchâtel, Switzerland; b Institute of Physics, University of Neuchâtel, rue Emile-Argand 11, CH-2000 Neuchâtel, Switzerland

**Keywords:** crystal structure, piperidine, hydrogen bonding, Hirshfeld surface analysis, energy frameworks

## Abstract

In the title compound, C_17_H_27_NO_2_, the piperidine ring has a chair conformation and is positioned normal to the benzene ring. In the crystal, mol­ecules are linked by C—H⋯O hydrogen bonds, forming chains propagating along the *c*-axis direction.

## Abstract 2

The title compound, 1-(2,5-di­meth­oxy­phen­yl)-2,2,6,6-tetra­methyl­piperidine, was synthesized as a side-product during the synthesis of the inter­mediate, methyl 3,6-dimeth­oxy-2-(2-meth­oxy-2-oxoeth­yl)benzoate, necessary for the total synthesis of the isocoumarin 5,8-dimeth­oxy-3-methyl-1*H*-isochromen-1-one.

## Chemical context   

**Figure 1:**
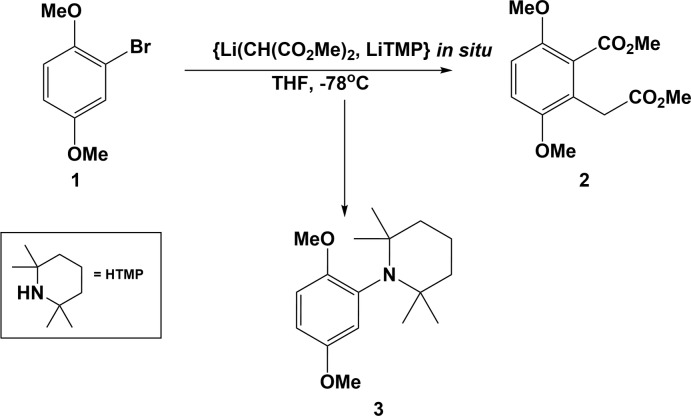
The reaction scheme resulting in the formation of the title compound, **3**.

During research on phytotoxins produced by the *Ceratocystis fimbriata* species (Tiouabi, 2005 ▸), the pathogenic agents responsible for the infections of plane, coffee and elm trees, analytical and spectroscopic studies enabled the isolation of a number of isocoumarins in small qu­anti­ties. In order to confirm their mol­ecular structures and especially to study their phytotoxicity and pathogenicity it was necessary to develop efficient methods for the total syntheses of these various isocoumarins. The title compound (**3**) was synthesized as a side product during the synthesis of the inter­mediate, methyl 3,6-dimeth­oxy-2-(2-meth­oxy-2-oxoeth­yl)benzoate (**2**) (see Fig. 1 ▸), necessary for the total synthesis of the isocoumarin 5,8-dimeth­oxy-3-methyl-1*H*-isochromen-1-one (Tiouabi, 2005 ▸).

## Structural commentary   

**Figure 2:**
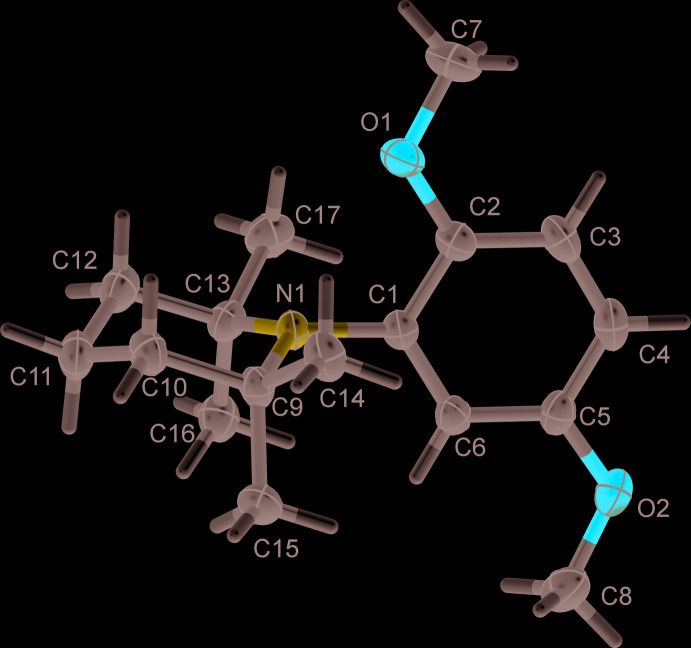
The mol­ecular structure of compound **3**, with atom labelling. Displacement ellipsoids are drawn at the 50% probability level.

The mol­ecular structure of the title compound, 1-(2,5-di­meth­oxy­phen­yl)-2,2,6,6-tetra­methyl­piperidine (**3**), is illus­trated in Fig. 2 ▸. The piperidine ring has a chair conformation with atoms N1 and C11 being displaced by −0.5171 (12) and 0.6876 (15) Å, respectively, from the mean plane of the remaining four C atoms (C9/C10/C12/C13). This mean plane is normal to the plane of the benzene ring (C1–C6), with a dihedral angle of 88.34 (9)°. Planes C2/O1/C7 and C5/O2/C8, involving the meth­oxy groups, are inclined to the benzene ring by 13.23 (15) and 10.45 (15)°, respectively.
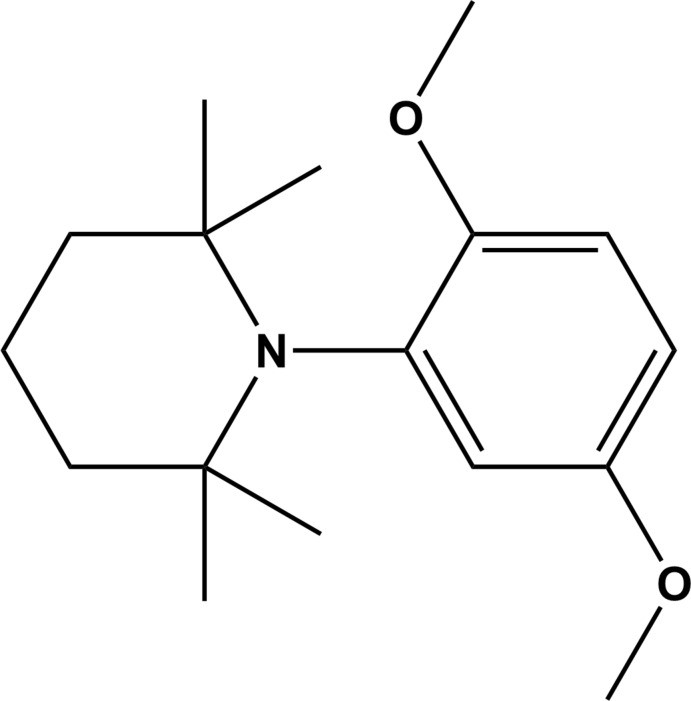


## Supra­molecular features   

**Figure 3:**
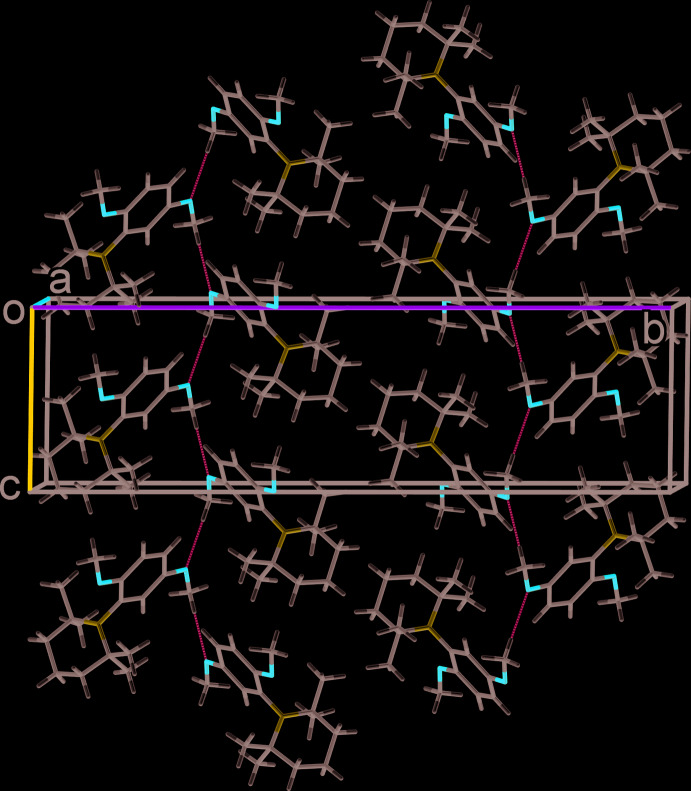
A view along the *a* axis of the crystal packing of compound **3**. The hydrogen bonds (Table 1 ▸) are shown as dashed lines.

In the crystal of **3**, mol­ecules related by the glide plane are linked by C—H⋯O hydrogen bonds, forming chains propagating along the *c*-axis direction (Fig. 3 ▸ and Table 1 ▸). There are no other significant inter­molecular inter­actions present in the crystal.

## Hirshfeld surface analysis and two-dimensional fingerprint plots   

The Hirshfeld surface analysis (Spackman & Jayatilaka, 2009 ▸) and the associated two-dimensional fingerprint plots (McKinnon *et al.*, 2007 ▸) were performed with *CrystalExplorer17.5* (Turner *et al.*, 2017 ▸). For an excellent explanation of the use of Hirshfeld surface analysis and other calculations, such as energy frameworks, to study the mol­ecular packing see the recent article by Tiekink and collaborators (Tan *et al.*, 2019 ▸). The Hirshfeld surface is colour-mapped with the normalized contact distance, *d*_norm_, from red (distances shorter than the sum of the van der Waals radii) through white to blue (distances longer than the sum of the van der Waals radii). The energy frameworks (Turner *et al.*, 2015 ▸; Tan *et al.*, 2019 ▸) are represented by cylinders joining the centroids of mol­ecular pairs using red, green and blue colour codes for the *E*_electrostatic_, *E*_dispersion_ and *E*_total_ energy components, respectively. The radius of the cylinder is proportional to the magnitude of the inter­action energy.

**Figure 4:**
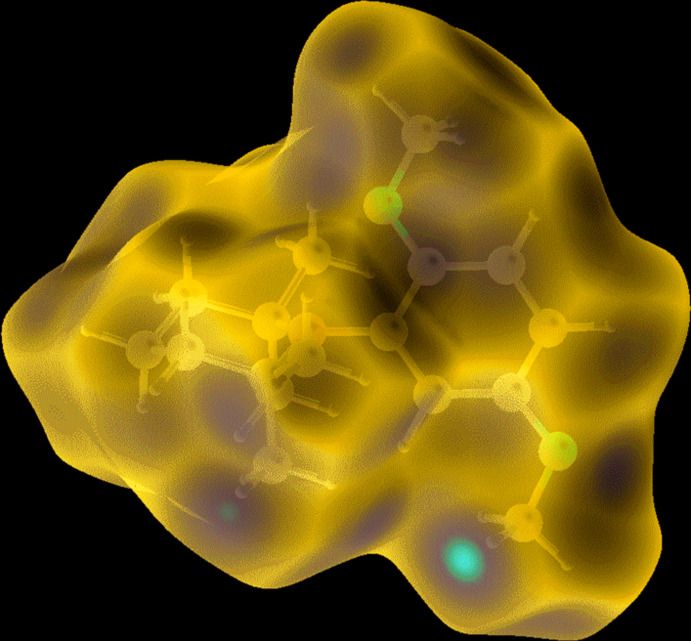
The Hirshfeld surface of compound **3** mapped over *d*_norm_, in the colour range −0.1434 to 1.2136 a.u..

**Figure 5:**
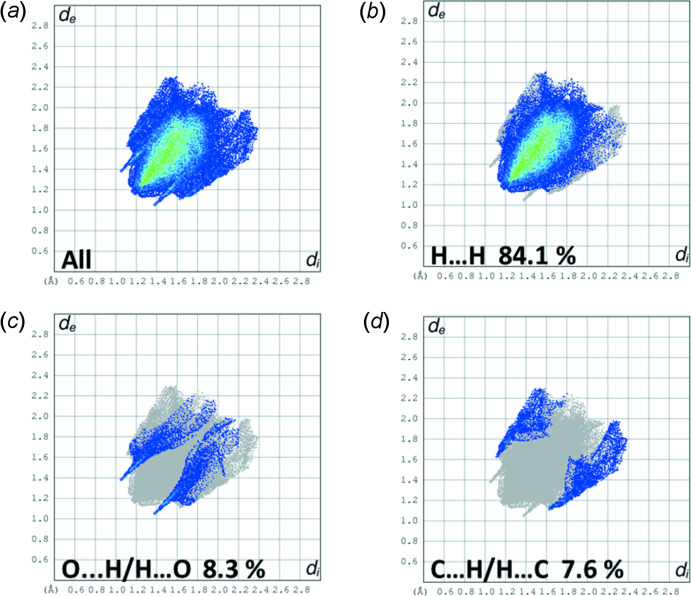
(*a*) The full two-dimensional fingerprint plot for compound **3**, and fingerprint plots delineated into (*b*) H⋯H at 84.1%, (*c*) O⋯H/H⋯O at 8.3% and (*d*) C⋯H/H⋯C at 7.6% contacts.

**Figure 6:**
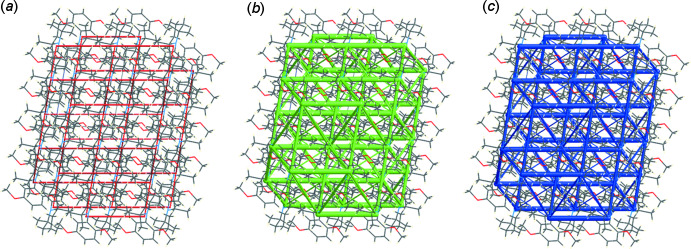
The energy frameworks viewed down the *b*-axis direction comprising (*a*) electrostatic potential forces, (*b*) dispersion forces and (*c*) total energy for a cluster about a reference mol­ecule of **3**. The energy frameworks were adjusted to the same scale factor of 80 with a cut-off value of 5 kJ mol^−1^ within 2 × 2 × 2 unit cells.

A view of the Hirshfeld surface of **3** mapped over *d*_norm_ is shown in Fig. 4 ▸. The short inter­atomic O⋯H/H⋯O contacts are indicated by the faint red spots. A full list of short inter­atomic contacts in the crystal of **3** are given in Table 2 ▸. The most significant contacts, apart from H⋯H contacts, are O⋯H and C⋯H contacts as confirmed by the two-dimensional fingerprint plots (Fig. 5 ▸). The principal inter­molecular contacts for **3**, are delineated into H⋯H at 84.1% (Fig. 5 ▸ *b*), O⋯H/H⋯O at 8.3% (Fig. 5 ▸ *c*) and C⋯H/H⋯C at 7.6% (Fig. 5 ▸ *d*) contacts. The inter­molecular contacts are therefore dominated by dispersion forces (H⋯H at 84.1%; Fig. 5 ▸ *b*). This is confirmed by the energy frameworks shown in Fig. 6 ▸. The energy frameworks were adjusted to the same scale factor of 80 with a cut-off value of 5 kJ mol^−1^ within 2 × 2 × 2 unit cells, and obtained using the wave function calculated at the HF/3-21G level of theory.

## Database survey   

A search of the Cambridge Structural Database (CSD, Version 5.41, last update March 2020; Groom *et al.*, 2016 ▸) for 1-(phen­yl)-2,2,6,6-tetra­methyl­piperidines gave 26 hits (see file S1 in the supporting information). A number of these structures involve heteroaryl and heterocyclic aluminium compounds, see for example CSD refcodes CEGLUY, CEGMF, CEGMEJ, CEGMIN, CEGMOT and CEGMUZ (Chen *et al.*, 2017 ▸). They also include a number of borohydride derivatives, see for example CSD refcodes JAKZON, JAKZUT, JALBAC and JALBEG (Chernichenko *et al.*, 2017 ▸). Only one compound has a meth­oxy substituent, *viz*. 1-(2-iodo-3-meth­oxy­phen­yl)-2,2,6,6-tetra­methyl­piperidine (VAPCUM; Crosbie *et al.*, 2012 ▸). In these eleven compounds, the piperidine ring has a chair conformation with the mean plane of the four planar C atoms being inclined to the plane of the benzene ring by dihedral angles varying from *ca* 83.0 to 90.0°. In compound **3** this dihedral angle is similar at 88.34 (9)°.

## Synthesis and crystallization   

The synthesis of compound **3** is illustrated in Fig. 1 ▸. It arises as a result of the condensation of 2-bromo-1,4-di­meth­oxy­benzene (**1**) with tetra­methyl­piper­idene (HTMP). It is a side product obtained during the synthesis of methyl 3,6-dimeth­oxy-2-(2-meth­oxy-2-oxoeth­yl)benzoate (**2**) (Tiouabi, 2005 ▸). Colourless rod-like crystals of **3** were obtained by slow evaporation at room temperature of a solution in acetone.There are no analytical or spectroscopic data available for compound **3**.

## Refinement details   

Crystal data, data collection and structure refinement details are summarized in Table 3 ▸. The hydrogen atoms were fixed geometrically (C—H = 0.95–0.99 Å) and allowed to ride on their parent atoms with *U*_iso_(H) = 1.5*U*_eq_(C-meth­yl) and 1.2*U*_eq_(C) for other H atoms.

## Supplementary Material

Crystal structure: contains datablock(s) I, Global. DOI: 10.1107/S2056989020005952/dj2006sup1.cif

Structure factors: contains datablock(s) I. DOI: 10.1107/S2056989020005952/dj2006Isup2.hkl

CSD search S1. DOI: 10.1107/S2056989020005952/dj2006sup3.pdf

Supporting information file. DOI: 10.1107/S2056989020005952/dj2006Isup4.cml

CCDC reference: 2000223

Additional supporting information:  crystallographic information; 3D view; checkCIF report

## Figures and Tables

**Table 1:** Hydrogen-bond geometry (Å, °)

*D*—H⋯*A*	*D*—H	H⋯*A*	*D*⋯*A*	*D*—H⋯*A*
C8—H8*B*⋯O2^i^	0.98	2.51	3.495 (2)	180

Symmetry code: (i) 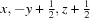.

**Table 2:** Short inter­atomic contacts (Å)*^*a*^* in the crystal of compound **3**

Atom1⋯Atom2	Length	Length − vdW
O2⋯H8*B* ^ii^	2.515	−0.205
C4⋯H15*C* ^iii^	2.830	−0.070
O2⋯H15*B* ^ii^	2.686	−0.034
C2⋯H8*A* ^iv^	2.918	0.018
C3⋯H14*C* ^iii^	2.977	0.077
H7*C*⋯H17*A* ^iv^	2.484	0.084
O1⋯H16*A* ^iv^	2.814	0.094

Note: (*a*) Calculated using *Mercury* (Macrae *et al.*, 2020 ▸). Symmetry codes: (ii) *x*, −*y* + 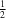, *z* − 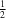; (iii) *x*, *y*, *z* − 1; (iv) *x* − 1, *y*, *z*.

**Table 3:** Experimental details

Crystal data	
Chemical formula	C_17_H_27_NO_2_
*M*_r_	277.39
Crystal system, space group	Monoclinic, *P*2_1_/*c*
Temperature (K)	173
*a*, *b*, *c* (Å)	6.8817 (10), 28.249 (4), 8.1369 (13)
β (°)	99.649 (12)
*V* (Å^3^)	1559.4 (4)
*Z*	4
Radiation type	Mo *K*α
μ (mm^−1^)	0.08
Crystal size (mm)	0.40 × 0.10 × 0.10

Data collection	
Diffractometer	STOE IPDS 2
No. of measured, independent and observed [*I* > 2σ(*I*)] reflections	10596, 2770, 1695
*R*_int_	0.085
(sin θ/λ)_max_ (Å^−1^)	0.599

Refinement	
*R*[*F* ^2^ > 2σ(*F* ^2^)], *wR*(*F* ^2^), *S*	0.037, 0.073, 0.83
No. of reflections	2770
No. of parameters	188
H-atom treatment	H-atom parameters constrained
Δρ_max_, Δρ_min_ (e Å^−3^)	0.13, −0.13

Computer programs: *X-AREA* and *X-RED32* (Stoe & Cie, 2005 ▸), *SHELXS97* (Sheldrick, 2008 ▸), *SHELXL2018/3* (Sheldrick, 2015 ▸), *PLATON* (Spek, 2020 ▸), *Mercury* (Macrae *et al.*, 2020 ▸) and *publCIF* (Westrip, 2010 ▸).

## supplementary crystallographic information

### Crystal data

**Table:**

C_17_H_27_NO_2_	*F*(000) = 608
*M**_r_* = 277.39	*D*_x_ = 1.182 Mg m^−^^3^
Monoclinic, *P*2_1_/*c*	Mo *K*α radiation, λ = 0.71073 Å
*a* = 6.8817 (10) Å	Cell parameters from 4940 reflections
*b* = 28.249 (4) Å	θ = 1.4–25.5°
*c* = 8.1369 (13) Å	µ = 0.08 mm^−^^1^
β = 99.649 (12)°	*T* = 173 K
*V* = 1559.4 (4) Å^3^	Rod, colourless
*Z* = 4	0.40 × 0.10 × 0.10 mm

### Data collection

**Table:**

STOE IPDS 2 diffractometer	1695 reflections with *I* > 2σ(*I*)
Radiation source: fine-focus sealed tube	*R*_int_ = 0.085
Plane graphite monochromator	θ_max_ = 25.2°, θ_min_ = 1.4°
φ + ω scans	*h* = −8→8
10596 measured reflections	*k* = −33→33
2770 independent reflections	*l* = −9→9

### Refinement

**Table:**

Refinement on *F*^2^	Secondary atom site location: difference Fourier map
Least-squares matrix: full	Hydrogen site location: inferred from neighbouring sites
*R*[*F*^2^ > 2σ(*F*^2^)] = 0.037	H-atom parameters constrained
*wR*(*F*^2^) = 0.073	*w* = 1/[σ^2^(*F*_o_^2^) + (0.0237*P*)^2^] where *P* = (*F*_o_^2^ + 2*F*_c_^2^)/3
*S* = 0.83	(Δ/σ)_max_ < 0.001
2770 reflections	Δρ_max_ = 0.13 e Å^−^^3^
188 parameters	Δρ_min_ = −0.13 e Å^−^^3^
0 restraints	Extinction correction: (*SHELXL2018/3*; Sheldrick, 2015), Fc^*^=kFc[1+0.001xFc^2^λ^3^/sin(2θ)]^-1/4^
Primary atom site location: structure-invariant direct methods	Extinction coefficient: 0.0127 (13)

### Special details

**Table:**

Geometry. All esds (except the esd in the dihedral angle between two l.s. planes) are estimated using the full covariance matrix. The cell esds are taken into account individually in the estimation of esds in distances, angles and torsion angles; correlations between esds in cell parameters are only used when they are defined by crystal symmetry. An approximate (isotropic) treatment of cell esds is used for estimating esds involving l.s. planes.

### Fractional atomic coordinates and isotropic or equivalent isotropic displacement parameters (Å^2^)

**Table:**

	*x*	*y*	*z*	*U*_iso_*/*U*_eq_	
O1	−0.15231 (15)	0.11464 (4)	0.48898 (14)	0.0359 (3)	
O2	0.47503 (17)	0.23289 (4)	0.44354 (15)	0.0389 (3)	
N1	0.17336 (17)	0.10517 (4)	0.72612 (15)	0.0239 (3)	
C1	0.1701 (2)	0.14010 (5)	0.59863 (18)	0.0240 (4)	
C2	0.0023 (2)	0.14458 (5)	0.4777 (2)	0.0274 (4)	
C3	−0.0050 (2)	0.17837 (6)	0.3529 (2)	0.0320 (4)	
H3	−0.120713	0.181465	0.271592	0.038*	
C4	0.1545 (2)	0.20752 (6)	0.3461 (2)	0.0326 (4)	
H4	0.148437	0.230424	0.259885	0.039*	
C5	0.3219 (2)	0.20347 (5)	0.4637 (2)	0.0286 (4)	
C6	0.3285 (2)	0.17020 (5)	0.58934 (19)	0.0264 (4)	
H6	0.443892	0.167808	0.671343	0.032*	
C7	−0.3053 (2)	0.11190 (7)	0.3485 (2)	0.0420 (5)	
H7C	−0.398183	0.086893	0.366432	0.063*	
H7B	−0.248449	0.104630	0.248721	0.063*	
H7A	−0.374711	0.142279	0.333365	0.063*	
C8	0.6384 (3)	0.23518 (6)	0.5748 (2)	0.0407 (5)	
H8A	0.703032	0.204187	0.588607	0.061*	
H8B	0.592985	0.244041	0.678454	0.061*	
H8C	0.732223	0.258909	0.548061	0.061*	
C9	0.1554 (2)	0.12313 (5)	0.89467 (19)	0.0269 (4)	
C10	0.0977 (2)	0.08181 (6)	0.9977 (2)	0.0342 (4)	
H10A	0.103522	0.092378	1.114443	0.041*	
H10B	−0.040201	0.072692	0.954118	0.041*	
C11	0.2287 (2)	0.03873 (6)	0.9958 (2)	0.0381 (5)	
H11A	0.365874	0.046683	1.046272	0.046*	
H11B	0.182345	0.012857	1.061593	0.046*	
C12	0.2215 (3)	0.02299 (6)	0.8172 (2)	0.0364 (4)	
H12B	0.084985	0.013326	0.770843	0.044*	
H12A	0.307259	−0.005084	0.815714	0.044*	
C13	0.2870 (2)	0.06129 (5)	0.7049 (2)	0.0294 (4)	
C14	−0.0136 (2)	0.15863 (6)	0.8748 (2)	0.0352 (4)	
H14C	−0.039727	0.167933	0.985055	0.053*	
H14B	−0.132032	0.144135	0.810935	0.053*	
H14A	0.022371	0.186676	0.815626	0.053*	
C15	0.3403 (2)	0.14765 (6)	0.9888 (2)	0.0361 (4)	
H15C	0.316036	0.157724	1.098873	0.054*	
H15B	0.371277	0.175361	0.925566	0.054*	
H15A	0.451420	0.125506	1.002183	0.054*	
C16	0.5112 (2)	0.06636 (6)	0.7429 (2)	0.0350 (4)	
H16C	0.572685	0.036915	0.713805	0.053*	
H16B	0.552574	0.072901	0.861923	0.053*	
H16A	0.552219	0.092519	0.677366	0.053*	
C17	0.2301 (2)	0.04478 (6)	0.5248 (2)	0.0387 (5)	
H17A	0.280543	0.067303	0.450547	0.058*	
H17B	0.086213	0.042978	0.495777	0.058*	
H17C	0.287008	0.013449	0.512256	0.058*	

### Atomic displacement parameters (Å^2^)

**Table:**

	*U*^11^	*U*^22^	*U*^33^	*U*^12^	*U*^13^	*U*^23^
O1	0.0285 (6)	0.0478 (7)	0.0295 (7)	−0.0066 (5)	−0.0007 (5)	0.0043 (6)
O2	0.0453 (7)	0.0328 (7)	0.0391 (7)	−0.0084 (6)	0.0085 (6)	0.0091 (6)
N1	0.0296 (7)	0.0217 (7)	0.0205 (7)	0.0021 (6)	0.0045 (6)	0.0027 (6)
C1	0.0292 (9)	0.0225 (8)	0.0210 (9)	0.0038 (7)	0.0058 (7)	−0.0006 (7)
C2	0.0276 (9)	0.0319 (9)	0.0233 (9)	0.0011 (7)	0.0063 (8)	−0.0023 (7)
C3	0.0353 (10)	0.0384 (10)	0.0218 (9)	0.0095 (8)	0.0032 (8)	0.0026 (8)
C4	0.0465 (11)	0.0275 (9)	0.0256 (9)	0.0094 (8)	0.0111 (9)	0.0059 (7)
C5	0.0362 (9)	0.0229 (8)	0.0283 (9)	0.0011 (7)	0.0098 (8)	0.0005 (7)
C6	0.0304 (9)	0.0230 (8)	0.0251 (8)	0.0027 (7)	0.0024 (7)	−0.0004 (7)
C7	0.0308 (10)	0.0594 (12)	0.0329 (10)	−0.0019 (9)	−0.0032 (8)	−0.0076 (9)
C8	0.0476 (11)	0.0392 (11)	0.0374 (11)	−0.0115 (9)	0.0136 (10)	−0.0044 (8)
C9	0.0334 (9)	0.0276 (9)	0.0197 (8)	0.0020 (7)	0.0045 (7)	0.0017 (7)
C10	0.0368 (10)	0.0400 (10)	0.0266 (9)	0.0011 (8)	0.0082 (8)	0.0080 (8)
C11	0.0397 (10)	0.0337 (10)	0.0409 (11)	0.0013 (8)	0.0068 (9)	0.0146 (8)
C12	0.0379 (10)	0.0248 (9)	0.0472 (11)	0.0019 (8)	0.0088 (9)	0.0065 (8)
C13	0.0318 (9)	0.0225 (8)	0.0340 (10)	0.0007 (7)	0.0063 (8)	0.0014 (7)
C14	0.0428 (10)	0.0355 (10)	0.0284 (10)	0.0095 (8)	0.0093 (8)	0.0006 (8)
C15	0.0427 (10)	0.0369 (10)	0.0270 (9)	−0.0030 (8)	0.0010 (8)	−0.0032 (8)
C16	0.0343 (9)	0.0309 (9)	0.0403 (10)	0.0064 (8)	0.0076 (8)	0.0036 (8)
C17	0.0426 (11)	0.0301 (9)	0.0437 (11)	0.0007 (8)	0.0085 (9)	−0.0079 (9)

### Geometric parameters (Å, º)

**Table:**

O1—C2	1.3743 (19)	C9—C15	1.536 (2)
O1—C7	1.4204 (18)	C10—C11	1.516 (2)
O2—C5	1.3732 (19)	C10—H10A	0.9900
O2—C8	1.416 (2)	C10—H10B	0.9900
N1—C1	1.4293 (19)	C11—C12	1.513 (2)
N1—C9	1.4867 (19)	C11—H11A	0.9900
N1—C13	1.4908 (19)	C11—H11B	0.9900
C1—C2	1.392 (2)	C12—C13	1.532 (2)
C1—C6	1.394 (2)	C12—H12B	0.9900
C2—C3	1.388 (2)	C12—H12A	0.9900
C3—C4	1.381 (2)	C13—C17	1.526 (2)
C3—H3	0.9500	C13—C16	1.529 (2)
C4—C5	1.374 (2)	C14—H14C	0.9800
C4—H4	0.9500	C14—H14B	0.9800
C5—C6	1.384 (2)	C14—H14A	0.9800
C6—H6	0.9500	C15—H15C	0.9800
C7—H7C	0.9800	C15—H15B	0.9800
C7—H7B	0.9800	C15—H15A	0.9800
C7—H7A	0.9800	C16—H16C	0.9800
C8—H8A	0.9800	C16—H16B	0.9800
C8—H8B	0.9800	C16—H16A	0.9800
C8—H8C	0.9800	C17—H17A	0.9800
C9—C14	1.524 (2)	C17—H17B	0.9800
C9—C10	1.528 (2)	C17—H17C	0.9800
			
C2—O1—C7	117.22 (13)	C9—C10—H10B	108.9
C5—O2—C8	117.75 (13)	H10A—C10—H10B	107.7
C1—N1—C9	116.13 (11)	C12—C11—C10	108.78 (14)
C1—N1—C13	115.76 (12)	C12—C11—H11A	109.9
C9—N1—C13	121.11 (11)	C10—C11—H11A	109.9
C2—C1—C6	118.02 (14)	C12—C11—H11B	109.9
C2—C1—N1	119.07 (14)	C10—C11—H11B	109.9
C6—C1—N1	122.90 (13)	H11A—C11—H11B	108.3
O1—C2—C3	122.57 (14)	C11—C12—C13	113.56 (14)
O1—C2—C1	117.18 (14)	C11—C12—H12B	108.9
C3—C2—C1	120.24 (15)	C13—C12—H12B	108.9
C4—C3—C2	120.51 (15)	C11—C12—H12A	108.9
C4—C3—H3	119.7	C13—C12—H12A	108.9
C2—C3—H3	119.7	H12B—C12—H12A	107.7
C5—C4—C3	120.13 (15)	N1—C13—C17	108.08 (12)
C5—C4—H4	119.9	N1—C13—C16	115.46 (12)
C3—C4—H4	119.9	C17—C13—C16	108.10 (14)
O2—C5—C4	115.91 (14)	N1—C13—C12	107.81 (13)
O2—C5—C6	124.65 (14)	C17—C13—C12	107.65 (13)
C4—C5—C6	119.42 (15)	C16—C13—C12	109.49 (13)
C5—C6—C1	121.67 (14)	C9—C14—H14C	109.5
C5—C6—H6	119.2	C9—C14—H14B	109.5
C1—C6—H6	119.2	H14C—C14—H14B	109.5
O1—C7—H7C	109.5	C9—C14—H14A	109.5
O1—C7—H7B	109.5	H14C—C14—H14A	109.5
H7C—C7—H7B	109.5	H14B—C14—H14A	109.5
O1—C7—H7A	109.5	C9—C15—H15C	109.5
H7C—C7—H7A	109.5	C9—C15—H15B	109.5
H7B—C7—H7A	109.5	H15C—C15—H15B	109.5
O2—C8—H8A	109.5	C9—C15—H15A	109.5
O2—C8—H8B	109.5	H15C—C15—H15A	109.5
H8A—C8—H8B	109.5	H15B—C15—H15A	109.5
O2—C8—H8C	109.5	C13—C16—H16C	109.5
H8A—C8—H8C	109.5	C13—C16—H16B	109.5
H8B—C8—H8C	109.5	H16C—C16—H16B	109.5
N1—C9—C14	107.86 (12)	C13—C16—H16A	109.5
N1—C9—C10	108.36 (12)	H16C—C16—H16A	109.5
C14—C9—C10	107.30 (14)	H16B—C16—H16A	109.5
N1—C9—C15	115.10 (13)	C13—C17—H17A	109.5
C14—C9—C15	108.04 (13)	C13—C17—H17B	109.5
C10—C9—C15	109.90 (13)	H17A—C17—H17B	109.5
C11—C10—C9	113.44 (14)	C13—C17—H17C	109.5
C11—C10—H10A	108.9	H17A—C17—H17C	109.5
C9—C10—H10A	108.9	H17B—C17—H17C	109.5
C11—C10—H10B	108.9		
			
C9—N1—C1—C2	−106.25 (16)	C1—N1—C9—C14	46.76 (17)
C13—N1—C1—C2	102.61 (16)	C13—N1—C9—C14	−163.75 (13)
C9—N1—C1—C6	73.77 (19)	C1—N1—C9—C10	162.62 (12)
C13—N1—C1—C6	−77.38 (18)	C13—N1—C9—C10	−47.89 (17)
C7—O1—C2—C3	13.5 (2)	C1—N1—C9—C15	−73.91 (16)
C7—O1—C2—C1	−166.84 (14)	C13—N1—C9—C15	75.58 (17)
C6—C1—C2—O1	−179.96 (14)	N1—C9—C10—C11	50.81 (17)
N1—C1—C2—O1	0.1 (2)	C14—C9—C10—C11	167.03 (14)
C6—C1—C2—C3	−0.3 (2)	C15—C9—C10—C11	−75.74 (17)
N1—C1—C2—C3	179.71 (14)	C9—C10—C11—C12	−57.94 (18)
O1—C2—C3—C4	−179.64 (15)	C10—C11—C12—C13	58.42 (18)
C1—C2—C3—C4	0.7 (2)	C1—N1—C13—C17	−46.30 (16)
C2—C3—C4—C5	−0.4 (2)	C9—N1—C13—C17	164.11 (13)
C8—O2—C5—C4	170.51 (15)	C1—N1—C13—C16	74.85 (17)
C8—O2—C5—C6	−11.2 (2)	C9—N1—C13—C16	−74.74 (18)
C3—C4—C5—O2	177.94 (15)	C1—N1—C13—C12	−162.40 (12)
C3—C4—C5—C6	−0.4 (2)	C9—N1—C13—C12	48.01 (17)
O2—C5—C6—C1	−177.36 (15)	C11—C12—C13—N1	−51.42 (17)
C4—C5—C6—C1	0.9 (2)	C11—C12—C13—C17	−167.79 (14)
C2—C1—C6—C5	−0.5 (2)	C11—C12—C13—C16	74.93 (18)
N1—C1—C6—C5	179.49 (14)		

### Hydrogen-bond geometry (Å, º)

**Table:**

*D*—H···*A*	*D*—H	H···*A*	*D*···*A*	*D*—H···*A*
C8—H8*B*···O2^i^	0.98	2.51	3.495 (2)	180

Symmetry code: (i) *x*, −*y*+1/2, *z*+1/2.
