# Supplementary material for: The crystal structure and Hirshfeld surface analysis of 1-(2,5-di­meth­oxy­phen­yl)-2,2,6,6-tetra­methyl­piperidine
Source: Acta Crystallogr E Crystallogr Commun. 2020 May 5;76(Pt 6):794–7. doi: 10.1107/S2056989020005952 (PMC7274006; doi:10.1107/S2056989020005952)

Search Overview

Search:	search3
Date/Time done:	Sun Apr 19 12:39:28 2020
Database(s):	CSD version 5.41 updates (Mar 2020)<br>CSD version 5.41 (November 2019)
Restriction Info:	No refcode restrictions applied
Filters:	None
Percentage Completed:	100%
Number of Hits:	26

Single query used. Search found structures that:

match

Query 1

Query 1

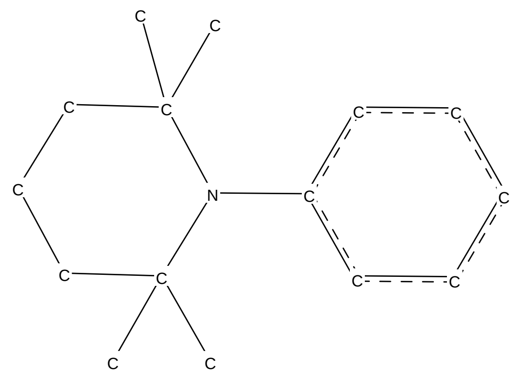

Search: search3 (Sun Apr 19 12:39:28 2020): Hits 1-4

BIRSAW

Reference: S.Olejnik, R.Allmann (1982) *Cryst.Struct.Commun.* ,**11,** 1083

Formula: $C_{15}\ H_{22}\ Br_1\ N_1$

Compound Name: N-(4-Bromophenyl)-2,2,6,6-tetramethylpiperidine

Space Group: P21/n
Space Group No.: 14

Cell: ***(Å,°)*** ***a*** 12.442(5) ***b*** 16.462(7) ***c*** 7.220(4)
α 90.00 β 90.12(2) γ 90.00

R-Factor (%): 7.65 ***Temperature(K):*** 295 ***Density(g/cm³):*** 1.330

Parameters

Fragment 1
ANG1 (A) 89.067

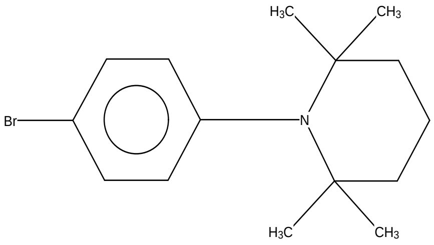

CEGLUY

Reference: Shimin Chen, Bin Li, Xiaoping Wang, Yanting Huang, Jiancheng Li, Hongping Zhu, Lili Zhao, G.Frenking, H.W.Roesky (2017) *Chem.-Eur.J.* ,**23,**13633

Formula: $C_{30}\ H_{48}\ Al_2\ N_2$

Compound Name: bis[2-(2,2,6,6-tetramethylpiperidin-1-yl)phenyl]-di-aluminium

Synonym: bis(μ-hydrido)-bis(2-(2,2,6,6-tetramethylpiperidin-1-yl)phenyl)-dihydrido-di-aluminium(iii)

Space Group: P21/c
Space Group No.: 14

Cell: ***(Å,°)*** ***a*** 7.946(0) ***b*** 23.553(0) ***c*** 7.807(0)
α 90.00 β 98.71(0) γ 90.00

R-Factor (%): 3.76 ***Temperature(K):*** 173 ***Density(g/cm³):*** 1.128

Parameters

Fragment 1
ANG1 (A) 89.309

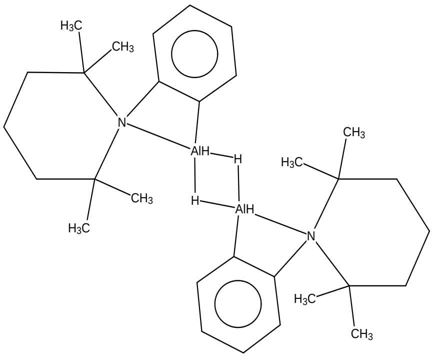

CEGMAF

Reference: Shimin Chen, Bin Li, Xiaoping Wang, Yanting Huang, Jiancheng Li, Hongping Zhu, Lili Zhao, G.Frenking, H.W.Roesky (2017) *Chem.-Eur.J.* ,**23,**13633

Formula: $C_{33}\ H_{38}\ Al_1\ N_3,0.25(C_7\ H_8)$

Compound Name: bis(1-methyl-1H-indol-3-yl)-(2-(2,2,6,6-tetramethylpiperidin-1-yl)phenyl)-aluminium(iii) toluene solvate

Space Group: P-1
Space Group No.: 2

Cell: ***(Å,°)*** ***a*** 11.400(0) ***b*** 15.924(0) ***c*** 17.963(0)
α 71.43(0) β 78.60(0) γ 71.16(0)

R-Factor (%): 7.50 ***Temperature(K):*** 173 ***Density(g/cm³):*** 1.203

Parameters

Fragment 1
ANG1 (A) 84.714

Fragment 2
ANG1 (A) 88.488

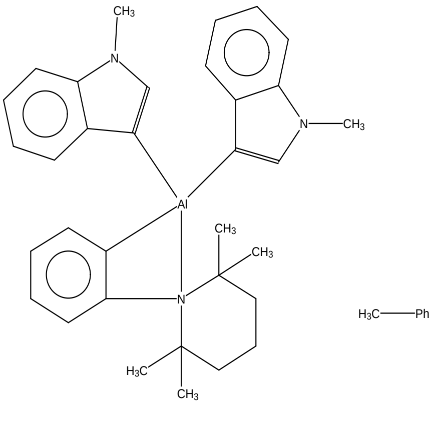

CEGMEJ

Reference: Shimin Chen, Bin Li, Xiaoping Wang, Yanting Huang, Jiancheng Li, Hongping Zhu, Lili Zhao, G.Frenking, H.W.Roesky (2017) *Chem.-Eur.J.* ,**23,**13633

Formula: $C_{35}\ H_{28}\ Al_1\ F_{10}\ N_1,1.5(C_7\ H_8)$

Compound Name: [phenylethynyl]-bis(pentafluorophenyl)-[2-(2,2,6,6-tetramethylpiperidinium-1-yl)phenyl]-aluminium(iii) toluene solvate

Space Group: C2/c
Space Group No.: 15

Cell: ***(Å,°)*** ***a*** 27.629(0) ***b*** 17.309(0) ***c*** 20.421(0)
α 90.00 β 124.49(0) γ 90.00

R-Factor (%): 6.39 ***Temperature(K):*** 173 ***Density(g/cm³):*** 1.350

Parameters

Fragment 1
ANG1 (A) 89.722

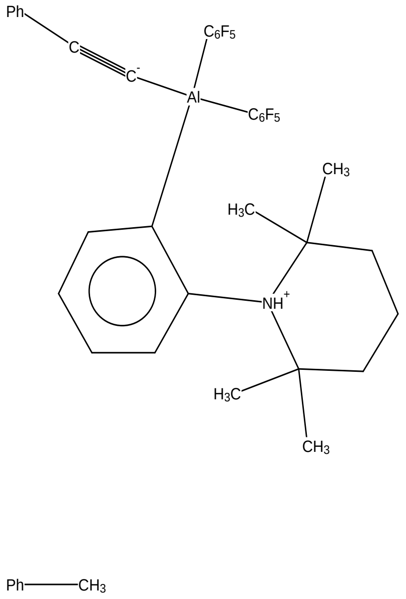

Search: search3 (Sun Apr 19 12:39:28 2020): Hits 5-8

CEGMIN

Reference: Shimin Chen, Bin Li, Xiaoping Wang, Yanting Huang, Jiancheng Li, Hongping Zhu, Lili Zhao, G.Frenking, H.W.Roesky (2017) *Chem.-Eur.J.* ,**23,**13633

Formula: $C_{26} H_{36} Al_1 N_1 Si_1$,0.5($C_7 H_8$)

Compound Name: (2-[2-(trimethylsilyl)ethen-2-id-1-yl]phenyl)-(2-(2,2,6,6-tetramethylpiperidin-1-yl)phenyl)-aluminium(iii) toluene solvate

Space Group:	Cc	***Cell:***	***a*** 19.134(0)	***b*** 10.944(0)	***c*** 25.686(0)
Space Group No.:	9	***(Å,°)***	α 90.00	β 96.10(0)	γ 90.00

R-Factor (%): 2.93 ***Temperature(K):*** 100 ***Density(g/cm^3):*** 1.152

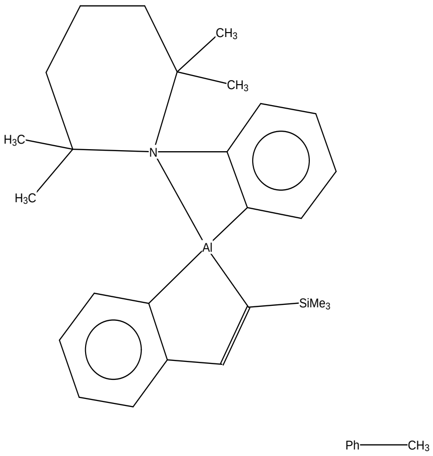

Parameters

Fragment 1
ANG1 (A) 89.455

Fragment 2
ANG1 (A) 89.554

CEGMOT

Reference: Shimin Chen, Bin Li, Xiaoping Wang, Yanting Huang, Jiancheng Li, Hongping Zhu, Lili Zhao, G.Frenking, H.W.Roesky (2017) *Chem.-Eur.J.* ,**23,**13633

Formula: $C_{32} H_{39} Al_1 N_2$

Compound Name: (2-[1-phenyl-2-(propan-2-ylazanidyl)ethenyl]phenyl)-(2-(2,2,6,6-tetramethylpiperidin-1-yl)phenyl)-aluminium(iii)

Space Group:	P21/n	***Cell:***	***a*** 15.725(0)	***b*** 10.000(0)	***c*** 18.352(0)
Space Group No.:	14	***(Å,°)***	α 90.00	β 110.95(0)	γ 90.00

R-Factor (%): 6.11 ***Temperature(K):*** 100 ***Density(g/cm^3):*** 1.180

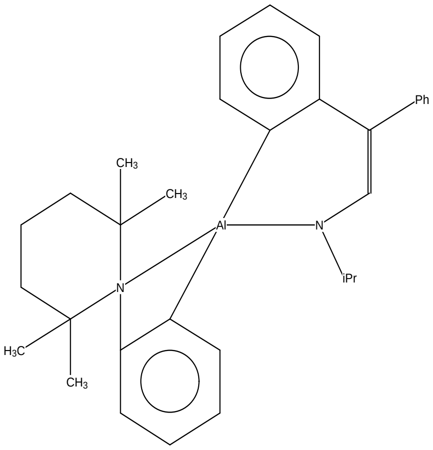

Parameters

Fragment 1
ANG1 (A) 83.736

CEGMUZ

Reference: Shimin Chen, Bin Li, Xiaoping Wang, Yanting Huang, Jiancheng Li, Hongping Zhu, Lili Zhao, G.Frenking, H.W.Roesky (2017) *Chem.-Eur.J.* ,**23,**13633

Formula: $C_{41} H_{49} Al_1 N_2$

Compound Name: ([2-(benzen-2-idyl)-2-phenylethenyl][2,6-di(propan-2-yl)phenyl]amido)-(2-(2,2,6,6-tetramethylpiperidin-1-yl)phenyl)-aluminium(iii)

Space Group:	P-1	***Cell:***	***a*** 10.972(0)	***b*** 18.250(0)	***c*** 18.382(1)
Space Group No.:	2	***(Å,°)***	α 74.97(0)	β 74.11(0)	γ 84.56(0)

R-Factor (%): 4.92 ***Temperature(K):*** 173 ***Density(g/cm^3):*** 1.160

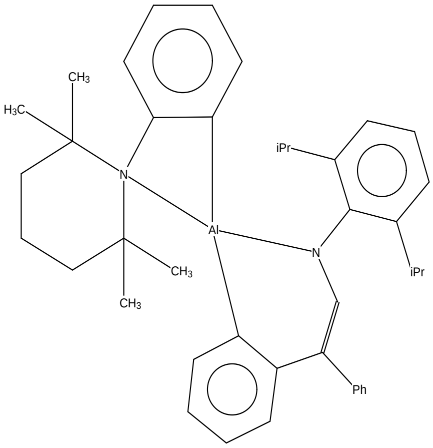

Parameters

Fragment 1
ANG1 (A) 83.079

Fragment 2
ANG1 (A) 86.092

CUVLOW

Reference: M.-A.Courtemanche, E.Rochette, M.-A.Legare, Wenhua Bi, F.-G.Fontaine (2016) *Dalton Trans.* ,**45,**6129

Formula: $C_{30} H_{45} B_1 N_2$

Compound Name: 1,1'-(boranediyldi-2,1-phenylene)bis(2,2,6,6-tetramethylpiperidine)

Space Group:	Pccn	***Cell:***	***a*** 22.415(1)	***b*** 7.755(0)	***c*** 15.540(0)
Space Group No.:	56	***(Å,°)***	α 90.00	β 90.00	γ 90.00

R-Factor (%): 5.06 ***Temperature(K):*** 150 ***Density(g/cm^3):*** 1.093

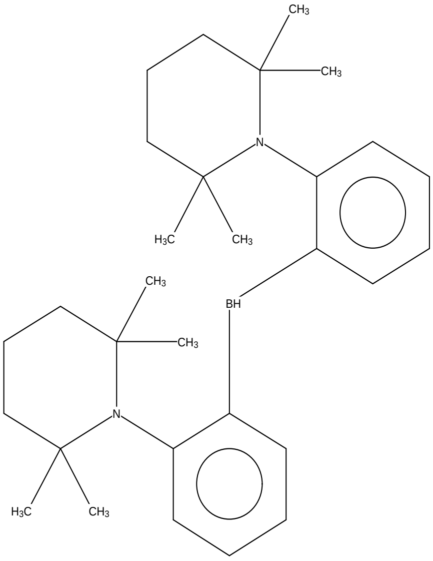

Parameters

Fragment 1
ANG1 (A) 84.029

Search: search3 (Sun Apr 19 12:39:28 2020): Hits 9-12

CUVLUC

Reference: M.-A.Courtemanche, E.Rochette, M.-A.Legare, Wenhua Bi, F.-G.Fontaine (2016) *Dalton Trans.* ,**45,**6129

Formula: $C_{30}\,H_{46}\,B_1\,Cl_1\,N_2,0.5(C_6\,H_6)$

Compound Name: Chloro(hydrido)(2-(2,2,6,6-tetramethylpiperidin-1-ium-1-yl)phenyl)(2-(2,2,6,6-tetramethylpiperidin-1-yl)phenyl)borate benzene solvate

Space Group:	P21/c	***Cell:***	***a*** 18.475(1)	***b*** 9.358(0)	***c*** 19.222(1)		
Space Group No.:	14	***(Å,°)***	α 90.00	β 111.68(0)	γ 90.00		
R-Factor (%):	3.89	***Temperature(K):***	150	***Density(g/cm³):***	1.118		

Parameters

Fragment 1
ANG1 (A) 88.536

Fragment 2
ANG1 (A) 87.392

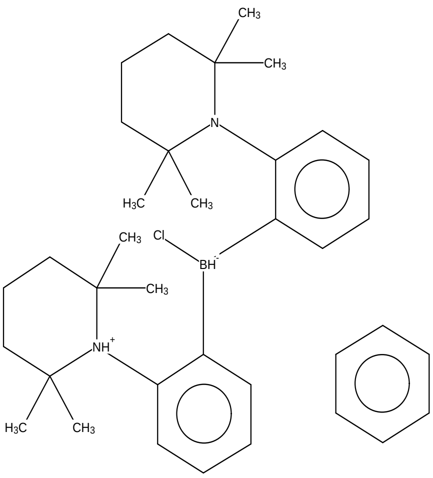

CUVMAJ

Reference: M.-A.Courtemanche, E.Rochette, M.-A.Legare, Wenhua Bi, F.-G.Fontaine (2016) *Dalton Trans.* ,**45,**6129

Formula: $C_{30}\,H_{44}\,B_1\,Cl_1\,N_2$

Compound Name: 1,1'-((Chloroboranediyl)di-2,1-phenylene)bis(2,2,6,6-tetramethylpiperidine)

Space Group:	Pccn	***Cell:***	***a*** 23.118(1)	***b*** 7.842(0)	***c*** 15.122(0)		
Space Group No.:	56	***(Å,°)***	α 90.00	β 90.00	γ 90.00		
R-Factor (%):	4.05	***Temperature(K):***	150	***Density(g/cm³):***	1.160		

Parameters

Fragment 1
ANG1 (A) 85.530

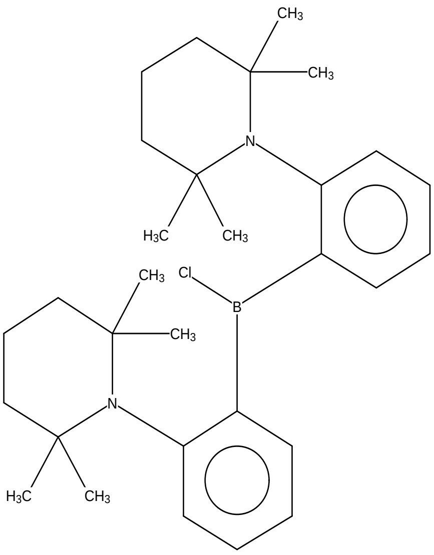

EJUXAK

Reference: M.-A.Legare, E.Rochette, J.L.Lavergne, N.Bouchard, F.-G.Fontaine (2016) *Chem.Commun.* ,**52,**5387

Formula: $C_{15}\,H_{24}\,B_1\,N_1\,O_2$

Compound Name: (2-(2,2,6,6-Tetramethylpiperidin-1-yl)phenyl)boronic acid

Space Group:	P21/n	***Cell:***	***a*** 13.854(0)	***b*** 7.816(0)	***c*** 15.243(1)		
Space Group No.:	14	***(Å,°)***	α 90.00	β 115.86(0)	γ 90.00		
R-Factor (%):	4.27	***Temperature(K):***	150	***Density(g/cm³):***	1.168		

Parameters

Fragment 1
ANG1 (A) 89.571

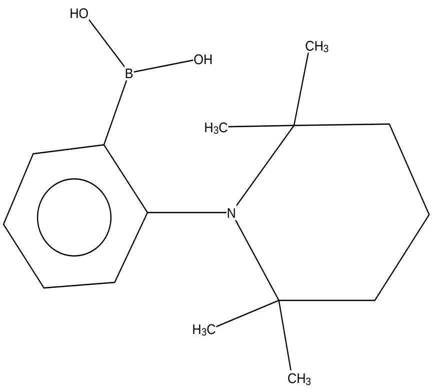

EJUXEO

Reference: M.-A.Legare, E.Rochette, J.L.Lavergne, N.Bouchard, F.-G.Fontaine (2016) *Chem.Commun.* ,**52,**5387

Formula: $C_{16}\,H_{26}\,B_1\,F_2\,N_1\,O_1$

Compound Name: Difluoro(methoxy)(2-(2,2,6,6-tetramethylpiperidinium-1-yl)phenyl)borate

Space Group:	P21/n	***Cell:***	***a*** 7.688(0)	***b*** 13.716(1)	***c*** 15.109(1)		
Space Group No.:	14	***(Å,°)***	α 90.00	β 98.44(0)	γ 90.00		
R-Factor (%):	4.47	***Temperature(K):***	150	***Density(g/cm³):***	1.252		

Parameters

Fragment 1
ANG1 (A) 87.464

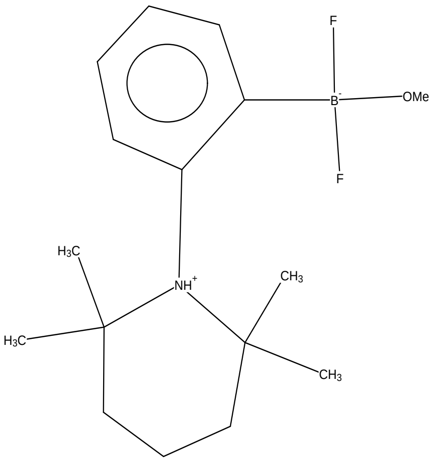

Search: search3 (Sun Apr 19 12:39:28 2020): Hits 13-16

EJUXIS

Reference: M.-A.Legare, E.Rochette, J.L.Lavergne, N.Bouchard, F.-G.Fontaine (2016) *Chem.Commun.* ,**52,**5387

Formula: $C_{15} H_{23} B_1 F_3 N_1$

Compound Name: Trifluoro(2-(2,2,6,6-tetramethylpiperidinium-1-yl)phenyl)borate

Space Group:	P21/c	***Cell:***	***a***	14.867(0)	***b***	7.633(0)	***c***	14.454(0)
Space Group No.:	14	***(Å,°)***	α	90.00	β	115.53(0)	γ	90.00

R-Factor (%): 4.42 ***Temperature(K):*** 150 ***Density(g/cm^3):*** 1.280

Parameters

Fragment 1
ANG1 (A) 88.970

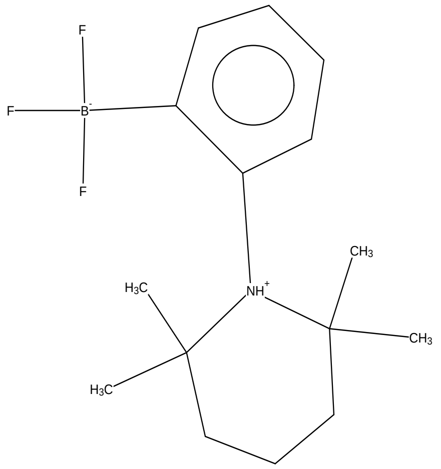

FOMWUC

Reference: Yilin Chen, Wenjun Jiang, Bin Li, Gang Fu, Shimin Chen, Hongping Zhu (2019) *Dalton Trans.* ,**48,**9152

Formula: $C_{70} H_{78} Al_2 N_2 P_2,2(C_6 D_6)$

Compound Name: bis(μ-1-(diphenylphosphino)-2-phenylethenyl)-dihydrido-bis(2-(2,2,6,6-tetramethylpiperidinyl)phenyl)-di-aluminium perdeuterobenzene solvate

Space Group:	P21/c	***Cell:***	***a***	13.992(0)	***b***	14.754(0)	***c***	18.776(0)
Space Group No.:	14	***(Å,°)***	α	90.00	β	92.47(0)	γ	90.00

R-Factor (%): 6.96 ***Temperature(K):*** 173 ***Density(g/cm^3):*** 1.046

Parameters

Fragment 1
ANG1 (A) 88.241

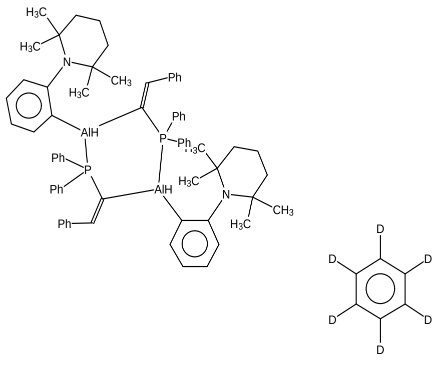

FOMYIS

Reference: Yilin Chen, Wenjun Jiang, Bin Li, Gang Fu, Shimin Chen, Hongping Zhu (2019) *Dalton Trans.* ,**48,**9152

Formula: $C_{27} H_{28} Al_1 N_1 S_2$

Compound Name: (2-(2,2,6,6-tetramethylpiperidinyl)phenyl)-bis(2-(2-thienyl)ethynyl)-aluminium

Space Group:	Pna21	***Cell:***	***a***	24.099(0)	***b***	8.701(0)	***c***	11.492(0)
Space Group No.:	33	***(Å,°)***	α	90.00	β	90.00	γ	90.00

R-Factor (%): 4.57 ***Temperature(K):*** 173 ***Density(g/cm^3):*** 1.261

Parameters

Fragment 1
ANG1 (A) 89.386

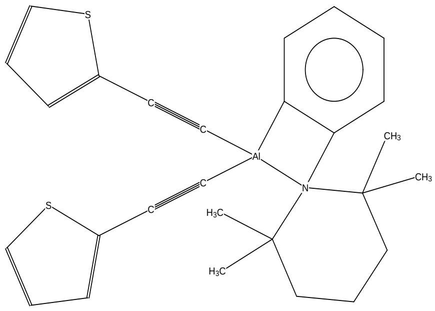

FOMYOY

Reference: Yilin Chen, Wenjun Jiang, Bin Li, Gang Fu, Shimin Chen, Hongping Zhu (2019) *Dalton Trans.* ,**48,**9152

Formula: $C_{44} H_{61} Al_1 N_2 Si_2$

Compound Name: (N-[2,6-di-isopropylphenyl]-1,1-dimethyl-N-[methyl(2-phenylethenyl)silyl]-1-phenylsilanamine)-(2-(2,2,6,6-tetramethylpiperidinyl)phenyl)-hydrido-aluminium

Space Group:	P21/c	***Cell:***	***a***	11.388(0)	***b***	19.676(0)	***c***	18.844(0)
Space Group No.:	14	***(Å,°)***	α	90.00	β	100.65(0)	γ	90.00

R-Factor (%): 6.09 ***Temperature(K):*** 173 ***Density(g/cm^3):*** 1.122

Parameters

Fragment 1
ANG1 (A) 89.238

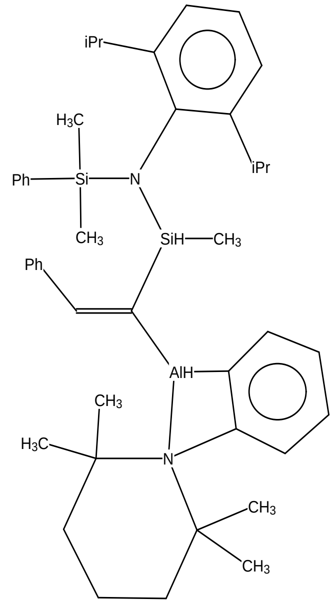

Search: search3 (Sun Apr 19 12:39:28 2020): Hits 17-20

FOMYUE

Reference: Yilin Chen, Wenjun Jiang, Bin Li, Gang Fu, Shimin Chen, Hongping Zhu (2019) *Dalton Trans.* ,**48,**9152

Formula: $C_{47}\ H_{58}\ Al_1\ N_1\ P_2\ Si_2$

Compound Name: (2-(2,2,6,6-tetramethylpiperidinyl)phenyl)-bis(2-(diphenylphosphino)-1-(dimethyl(hydrido)silyl)ethenyl)-aluminium

Space Group:	P21/c	***Cell:***	***a***	11.354(1)	***b***	17.484(1)	***c***	22.700(1)
Space Group No.:	14	***(Å,°)***	α	90.00	β	98.93(0)	γ	90.00

R-Factor (%): 7.00 ***Temperature(K):*** 173 ***Density(g/cm^3):*** 1.167

Parameters

Fragment 1
ANG1 (A) 88.393

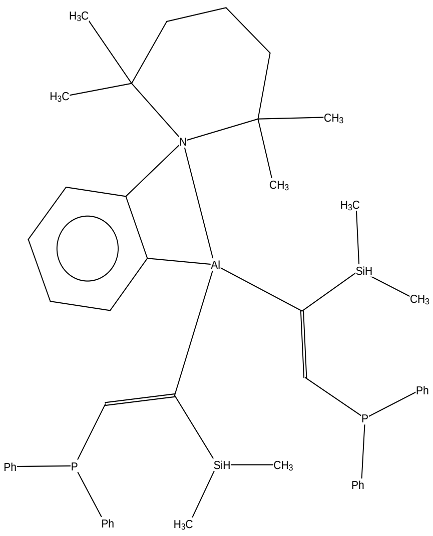

IMIVEH

Reference: K.Chernichenko, M.Lindqvist, B.Kotai, M.Nieger, K.Sorochkina, I.Papai, Timo Repo (2016) *J.Am.Chem.Soc.* ,**138,**4860

Formula: $C_{39}\ H_{40}\ B_1\ N_1$

Compound Name: 5-([1,1'-Biphenyl]-2-yl)-5-(2-(2,2,6,6-tetramethylpiperidin-1-ium-1-yl)phenyl)-5H-dibenzo[b,d]borol-5-uide

Space Group:	P21/c	***Cell:***	***a***	19.180(0)	***b***	18.535(0)	***c***	18.409(0)
Space Group No.:	14	***(Å,°)***	α	90.00	β	115.64(0)	γ	90.00

R-Factor (%): 5.83 ***Temperature(K):*** 123 ***Density(g/cm^3):*** 1.201

Parameters

Fragment 1
ANG1 (A) 84.740

Fragment 2
ANG1 (A) 85.317

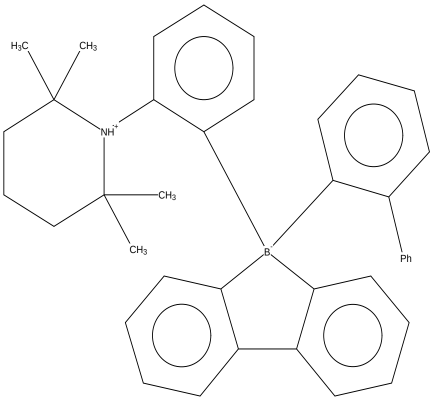

JAKZON

Reference: K.Chernichenko, B.Kotai, M.Nieger, S.Heikkinen, I.Papai, T.Repo (2017) *Dalton Trans.* ,**46,**2263

Formula: $C_{15}\ H_{23}\ B_1\ Cl_3\ N_1$

Compound Name: trichloro(2-(2,2,6,6-tetramethylpiperidin-1-ium-1-yl)phenyl)borate

Space Group:	Cc	***Cell:***	***a***	7.867(1)	***b***	15.592(2)	***c***	14.279(2)
Space Group No.:	9	***(Å,°)***	α	90.00	β	104.89(2)	γ	90.00

R-Factor (%): 3.41 ***Temperature(K):*** 123 ***Density(g/cm^3):*** 1.313

Parameters

Fragment 1
ANG1 (A) 88.630

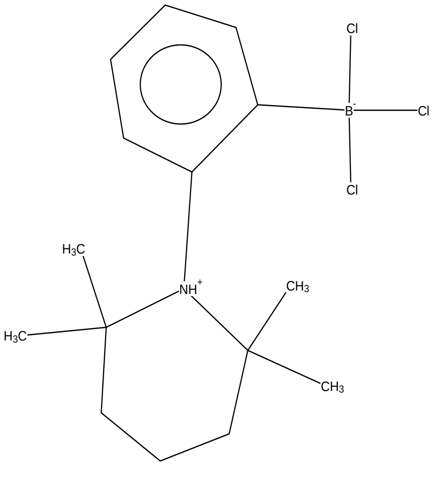

JAKZUT

Reference: K.Chernichenko, B.Kotai, M.Nieger, S.Heikkinen, I.Papai, T.Repo (2017) *Dalton Trans.* ,**46,**2263

Formula: $C_{21}\ H_{23}\ B_1\ Cl_2\ F_5\ N_1$

Compound Name: dichloro(pentafluorophenyl)(2-(2,2,6,6-tetramethylpiperidin-1-ium-1-yl)phenyl)borate

Space Group:	Pbca	***Cell:***	***a***	14.123(1)	***b***	15.558(1)	***c***	19.000(2)
Space Group No.:	61	***(Å,°)***	α	90.00	β	90.00	γ	90.00

R-Factor (%): 4.05 ***Temperature(K):*** 123 ***Density(g/cm^3):*** 1.483

Parameters

Fragment 1
ANG1 (A) 89.071

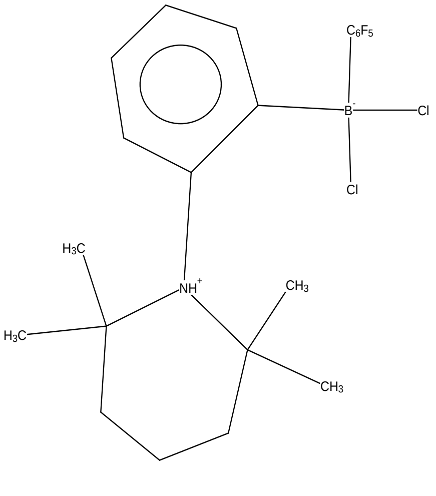

Search: search3 (Sun Apr 19 12:39:28 2020): Hits 21-24

JALBAC

Reference: K.Chernichenko, B.Kotai, M.Nieger, S.Heikkinen, I.Papai, T.Repo (2017) *Dalton Trans.* ,**46,**2263

Formula: $C_{15}\,H_{24}\,B_1\,Cl_2\,N_1$

Compound Name: dichloro(hydrido)(2-(2,2,6,6-tetramethylpiperidin-1-ium-1-yl)phenyl) borate

Space Group:	Cmc21	***Cell:***	***a***	11.185(0)	***b***	11.328(0)	***c***	12.598(0)
Space Group No.:	36	***(Å,°)***	α	90.00	β	90.00	γ	90.00

R-Factor (%): 2.25 ***Temperature(K):*** 123 ***Density(g/cm^3):*** 1.249

Parameters

Fragment 1
ANG1 (A) 90.000

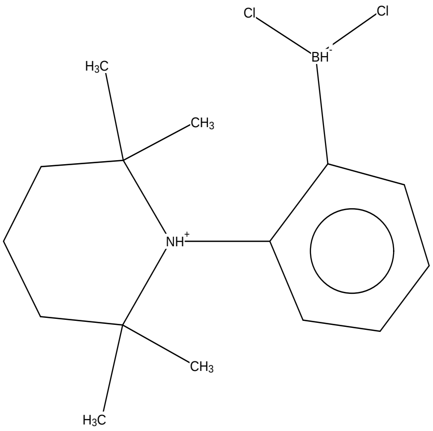

JALBEG

Reference: K.Chernichenko, B.Kotai, M.Nieger, S.Heikkinen, I.Papai, T.Repo (2017) *Dalton Trans.* ,**46,**2263

Formula: $C_{21}\,H_{24}\,B_1\,Cl_1\,F_5\,N_1$

Compound Name: chloro(hydrido)(pentafluorophenyl)(2-(2,2,6,6-tetramethylpiperidin-1-ium-1-yl)phenyl)borate

Space Group:	P212121	***Cell:***	***a***	10.890(0)	***b***	12.306(0)	***c***	15.297(1)
Space Group No.:	19	***(Å,°)***	α	90.00	β	90.00	γ	90.00

R-Factor (%): 3.15 ***Temperature(K):*** 123 ***Density(g/cm^3):*** 1.399

Parameters

Fragment 1
ANG1 (A) 88.340

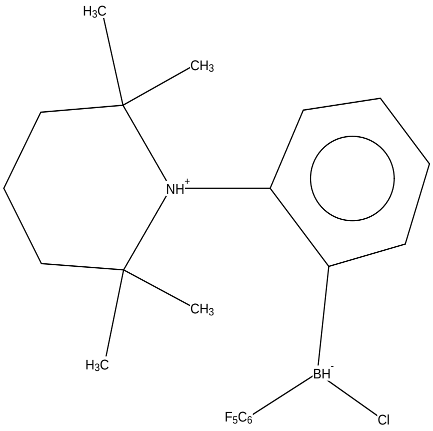

NEWKAD

Reference: K.Chernichenko, M.Nieger, M.Leskela, T.Repo (2012) *Dalton Trans.* ,**41,**9029

Formula: $C_{27}\,H_{24}\,B_1\,F_{10}\,N_1,0.5(C_6\,D_6)$

Compound Name: bis(Perfluorophenyl)(2-(2,2,6,6-tetramethylpiperidin-1-ium-1-yl)phenyl) hydridoborate deuterobenzene solvate

Space Group:	P-1	***Cell:***	***a***	10.944(1)	***b***	11.974(1)	***c***	12.513(1)
Space Group No.:	2	***(Å,°)***	α	117.39(1)	β	98.29(1)	γ	106.57(1)

R-Factor (%): 4.36 ***Temperature(K):*** 123 ***Density(g/cm^3):*** 1.523

Parameters

Fragment 1
ANG1 (A) 84.829

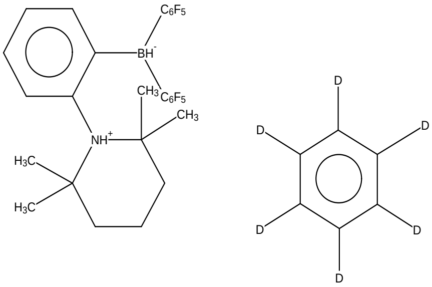

NEWKEH

Reference: K.Chernichenko, M.Nieger, M.Leskela, T.Repo (2012) *Dalton Trans.* ,**41,**9029

Formula: $C_{27}\,H_{22}\,B_1\,F_{10}\,N_1$

Compound Name: 1-(2-(bis(Pentafluorophenyl)boryl)phenyl)-2,2,6,6-tetramethylpiperidine

Space Group:	P21/c	***Cell:***	***a***	7.891(0)	***b***	17.344(1)	***c***	17.867(1)
Space Group No.:	14	***(Å,°)***	α	90.00	β	101.33(0)	γ	90.00

R-Factor (%): 5.79 ***Temperature(K):*** 123 ***Density(g/cm^3):*** 1.555

Parameters

Fragment 1
ANG1 (A) 72.055

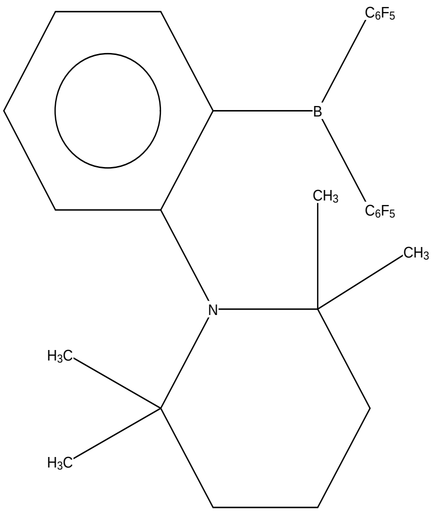

Search: search3 (Sun Apr 19 12:39:28 2020): Hits 25-26

TORREZ

Reference:	K.Chernichenko, B.Kotai, I.Papai, V.Zhivonitko, M.Nieger, M.Leskela, Timo Repo (2015) *Angew.Chem.,Int.Ed.* ,**54,**1749
Formula:	$C_{30} H_{48} B_2 N_2$
Compound Name:	bis(μ_2-Hydrido)-bis(hydrido(2-(2,2,6,6-tetramethylpiperidinyl)phenyl) borane)

Space Group:	P21/n	***Cell:***	***a*** 12.841(0)	***b*** 7.575(0)	***c*** 14.910(0)		
Space Group No.:	14	***(Å,°)***	α 90.00	β 109.94(0)	γ 90.00		
R-Factor (%):	4.55	***Temperature(K):***	123	***Density(g/cm3):***	1.117		

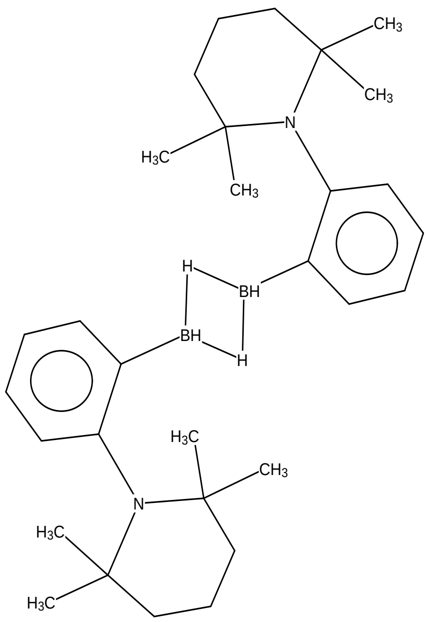

Parameters

Fragment 1
ANG1 (A) 83.854

VAPCUM

Reference:	E.Crosbie, A.R.Kennedy, R.E.Mulvey, S.D.Robertson (2012) *Dalton Trans.* ,**41,**1832
Formula:	$C_{16} H_{24} I_1 N_1 O_1$
Compound Name:	1-(2-Iodo-3-methoxyphenyl)-2,2,6,6-tetramethylpiperidine

Space Group:	Fdd2	***Cell:***	***a*** 12.882(0)	***b*** 59.071(1)	***c*** 8.433(0)		
Space Group No.:	43	***(Å,°)***	α 90.00	β 90.00	γ 90.00		
R-Factor (%):	2.12	***Temperature(K):***	123	***Density(g/cm3):***	1.545		

Parameters

Fragment 1
ANG1 (A) 86.995

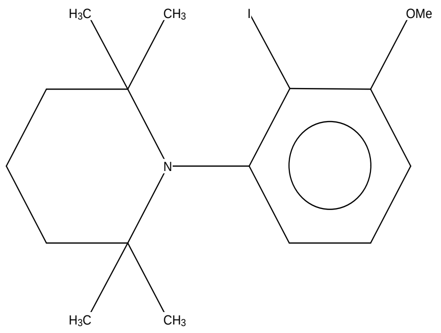

Supplement: Supplementary file 3 [file e-76-00794-sup3.pdf]
